# Hubble Meets Webb: Image-to-Image Translation in Astronomy

**DOI:** 10.3390/s24041151

**Published:** 2024-02-09

**Authors:** Vitaliy Kinakh, Yury Belousov, Guillaume Quétant, Mariia Drozdova, Taras Holotyak, Daniel Schaerer, Slava Voloshynovskiy

**Affiliations:** 1 Department of Computer Science, University of Geneva, 1227 Carouge, Switzerland; vitaliy.kinakh@unige.ch (V.K.); yury.belousov@unige.ch (Y.B.); guillaume.quetant@unige.ch (G.Q.); mariia.drozdova@unige.ch (M.D.); taras.holotyak@unige.ch (T.H.); 2 Department of Astronomy, University of Geneva, 1290 Versoix, Switzerland; daniel.schaerer@unige.ch

**Keywords:** image-to-image translation, denoising diffusion probabilistic models, uncertainty estimation, satellite image generation, image registration

## Abstract

This work explores the generation of James Webb Space Telescope (JWSP) imagery via image-to-image translation from the available Hubble Space Telescope (HST) data. Comparative analysis encompasses the Pix2Pix, CycleGAN, TURBO, and DDPM-based Palette methodologies, assessing the criticality of image registration in astronomy. While the focus of this study is not on the scientific evaluation of model fairness, we note that the techniques employed may bear some limitations and the translated images could include elements that are not present in actual astronomical phenomena. To mitigate this, uncertainty estimation is integrated into our methodology, enhancing the translation’s integrity and assisting astronomers in distinguishing between reliable predictions and those of questionable certainty. The evaluation was performed using metrics including MSE, SSIM, PSNR, LPIPS, and FID. The paper introduces a novel approach to quantifying uncertainty within image translation, leveraging the stochastic nature of DDPMs. This innovation not only bolsters our confidence in the translated images but also provides a valuable tool for future astronomical experiment planning. By offering predictive insights when JWST data are unavailable, our approach allows for informed preparatory strategies for making observations with the upcoming JWST, potentially optimizing its precious observational resources. To the best of our knowledge, this work is the first attempt to apply image-to-image translation for astronomical sensor-to-sensor translation.

## 1. Introduction

In this paper, we explore the problem of predicting the visible sky images captured by the James Webb Space Telescope (JWST), hereafter referred to as ‘Webb’ [1], using the available data from the Hubble Space Telescope (HST), hereinafter called ‘Hubble’ [2]. There is much interest in this type of problem in fields such as astrophysics, astronomy, and cosmology, encompassing a variety of data types and sources. This includes the translation of observations of galaxies in visible light [3] and predictions of dark matter [4]. The data registered from different sources may be acquired at different times, by different sensors, in different bands, with different resolutions, sensitivities, and levels of noise. The exact underlying mathematical model for transforming data between these sources is very complex and largely unknown. Thus, we will try to address this problem based on an image-to-image translation approach.

Despite the great success of image-to-image translation in computer vision, its adoption in the astrophysics community has been limited, even though there is a lot of data available for such tasks that might enable sensor-to-sensor translation, conversion between different spectral bands, and adaptation among various satellite systems.

Before the launch of missions such as Euclid [5], the radio telescope Square Kilometre Array [6], and others, there has been a significant interest in advancing image-to-image translation techniques for astronomical data to: (i) enable efficient mission planning due to the high complexity and cost of exhaustive space exploration, allowing for the prioritization of specific space regions using existing data; and (ii) generate sufficient synthetic data for machine learning (ML) analysis as soon as the first real images from new imaging missions are available in adequate quantities.

**Figure 1:**
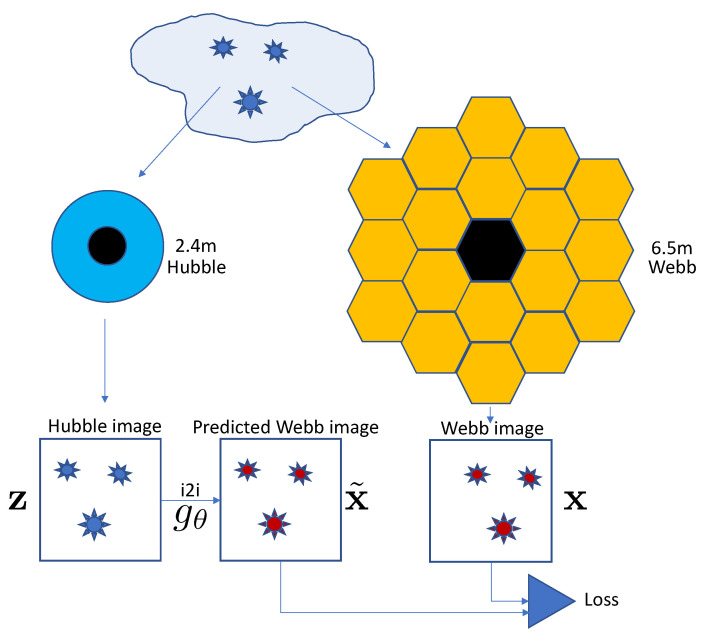
Image-to-image astronomical setup under study. Given two imaging systems, Hubble and Webb, characterized by different bands, resolutions, orbits, and time of image acquisition, the problem is to predict the Webb images $\tilde{x}$ as close as possible to the original Webb images x from the Hubble ones z using a learnable model $g_{\theta }$. The considered setup is paired but is characterized by inaccurate geometrical synchronization between the paired images.

We focus on the images collected by both the Hubble and the Webb telescopes, taken at different times, as illustrated in Figure 1. Thus, we present our work as a proof-of-concept for image-to-image translation, aiming to predict Webb telescope images using those from Hubble. This technique, once validated, could inform the planning of future missions and experiments by enabling the prediction of Webb telescope observations from existing Hubble data.

We assume that, despite the time lapse between Hubble’s and Webb’s data acquisition, the astronomical scenes of interest have remained relatively stable, conforming to the slow-changing physics of the observed phenomena. However, there is a substantial disparity in the imaging technologies of the two telescopes, affecting not only resolution and signal-to-noise ratio but also the visual representation of the phenomena due to different underlying physical principles and the images being taken at various wavelengths.

Our study reveals that Hubble and Webb data are typically dis-synchronized by approximately 3–5 pixels, a discrepancy mainly attributed to synchronization with respect to celestial coordinates during Webb’s data pre-processing and differing resolutions. Although this misalignment is subtle to the naked eye, we found that it significantly impairs the accuracy of paired image-to-image translation, highlighting the critical need for precise data alignment. To address this problem, we introduce two synchronization methods using computer vision keypoints and descriptors: (a) global synchronization applies a single affine transformation to the entire image; (b) local synchronization divides the image into patches and computes individual affine transformations for each patch. We compare the impact on the performance of image-to-image translation when using these synchronization methods against provided synchronization with respect to celestial coordinates.

We compare several types of image-to-image translation methods: (i) fully paired methods such as Pix2Pix [7] and their variations; (ii) fully unpaired methods such as CycleGAN [8]; (iii) hybrid methods that can be used for both fully paired setups, fully unpaired setups, or setups where part of the data is paired, and part of the data is unpaired, as advocated by the TURBO approach [9]; (iv) denoising diffusion probabilistic models (DDPM) [10] based image-to-image translation method Palette [11]. We investigate the influence of pairing and different types of synchronization for the above methods. We demonstrate that paired methods produce results superior to unpaired ones. At the same time, the paired methods Pix2Pix and TURBO are subject to the accuracy of synchronization. Local synchronization produces the most accurate translation results, according to several metrics of performance.

Furthermore, we show that there is a high potential for uncertainty in the estimation when using DDPM models for image-to-image translation since they can produce multiple outputs for one input. This stochastic translation enabled us to establish the regions that appear to be very stable in each run and the ones that are characterized by high variability.

In summary, we run experiments for image-to-image translation on non-synchronized, globally synchronized, and locally synchronized Hubble–Webb pairs. We report the results using multiple metrics: MSE, SSIM [12], PSNR, LPIPS [13], and FID [14]. We use computer vision-based metrics since we are working with telescope images represented as RGB images.

The main focus of this paper is not on the scientific inquiry into the fairness of predictive models. We acknowledge that our results, generated through the image-to-image translation technique, are subject to limitations inherent to such approaches. The data and methods utilized may not be exhaustive or infallible, and the results should therefore be interpreted with caution, as they are not immune to inaccuracies and may contain hallucinated elements which do not correspond to real astronomical phenomena.

Therefore, to enhance the integrity of the image-to-image translation provided in this study, we incorporate uncertainty estimation into our methodology. This feature is designed to assist astronomers by delineating areas within the translated images where the model’s predictions are reliable from those where the certainty of prediction remains questionable. Such delineation is crucial in guiding astronomers to discern between regions of high confidence and those that require further scrutiny or could potentially mislead them.

The proposed approach, with its ability to estimate uncertainty, may serve as an instrumental tool for planning future astronomical experiments. In scenarios where observational data from the Webb telescope are not yet available, our model can offer predictive insights based on existing Hubble Space Telescope data. This capability acts as a provisional glimpse into the future, enabling researchers to strategize upcoming observations with the Webb telescope, potentially optimizing the allocation of its valuable observational time.

Our contributions include: (i) the introduction of image-based synchronization for astrophysics data in view of image-to-image translation problems; (ii) a comparison of the image-to-image translation methods for Hubble to Webb translation, and a study of the effect of synchronization on different models; (iii) the introduction of an innovative way of uncertainty estimation in probabilistic inverse solvers or translation methods based on denoising diffusion probabilistic models. In summary, **our main contribution** is: the demonstration of the potential of using deep learning-based image-to-image translation in astronomical imaging, exemplified by Hubble to Webb image translation.

## 2. Related Work

### 2.1. Comparison between Webb and Hubble Telescopes

**Figure 2:**
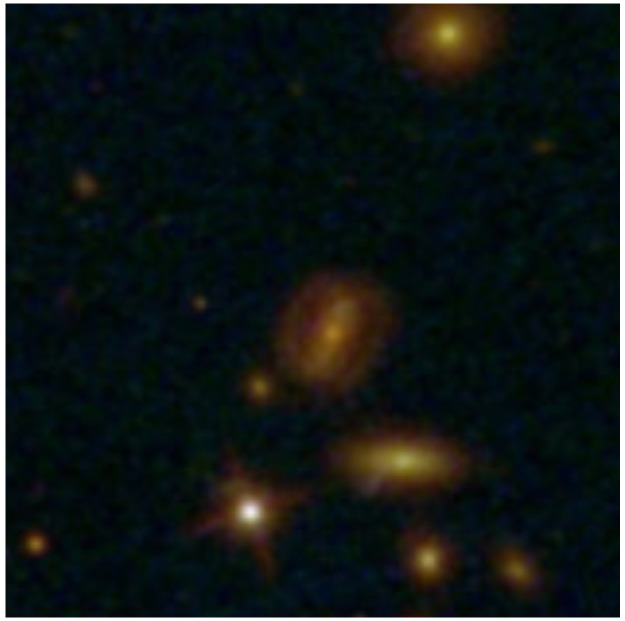
Hubble photo of Galaxy Cluster SMACS 0723 [17].

**Figure 3:**
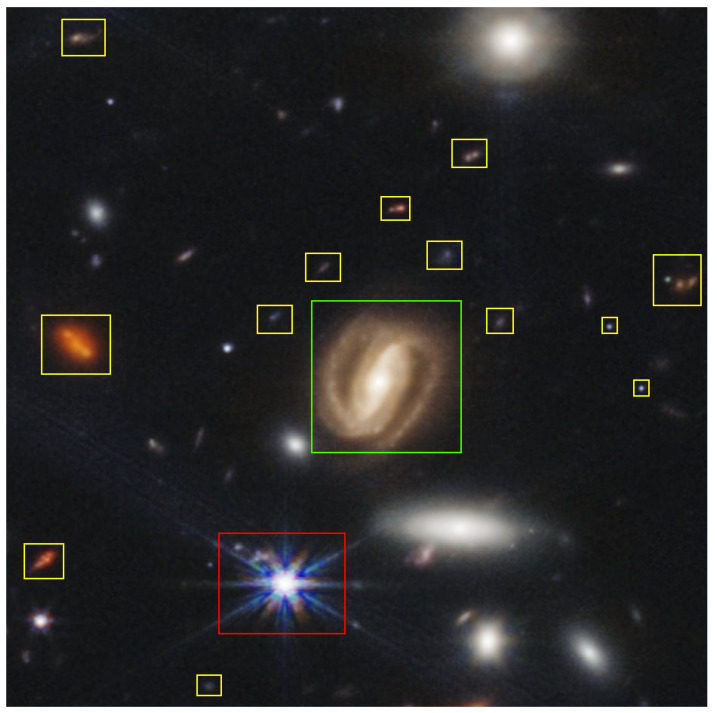
Webb image of Galaxy Cluster SMACS 0723 [18].

In Figure 2 and Figure 3, the same part of the sky captured by the Hubble and Webb telescopes is shown in the RGB format. The main differences between the Hubble and Webb telescopes are: (i) **Spatial resolution**—The Webb telescope, featuring a 6.5-m primary mirror, offers superior resolution compared to Hubble’s 2.4-m mirror, which is particularly noticeable in infrared observations [15]. This enables Webb to capture images of objects up to 100 times fainter than Hubble, as evident in the central spiral galaxy in Figure 3.

(ii) **Wavelength coverage**— Hubble, optimized for ultraviolet and visible light (0.1 to 2.5 microns), contrasts with Webb’s focus on infrared wavelengths (0.6 to 28.5 microns) [16]. While this differentiation allows Webb to observe more distant and fainter celestial objects, including the earliest stars and galaxies, it is crucial to note that the IR emission captured by Webb differs inherently from the UV or visible light observed by Hubble. The distinction is not solely in the resolution or sensitivity between the Hubble Space Telescope (HST) and the James Webb Space Telescope (JWST) but also in the varying absorption of light by dust within different galaxy types. However, our proposed image-to-image translation method does not aim to delve into these observational differences. Instead, our focus is to explore whether image-to-image translation can effectively simulate Webb telescope imagery based on the existing data from Hubble. This approach seeks to leverage the available Hubble data to anticipate and interpret the observations that Webb might deliver, without directly analyzing the spectral and compositional differences between the images captured by the two telescopes.

(iii) **Light-collecting capacity**—Webb’s substantially larger mirror provides over six times the light-collecting area compared to Hubble, essential for studying longer, dimmer wavelengths of light from distant, redshifted objects [15]. This is exemplified in Webb’s images, which reveal smaller galaxies and structures not visible in Hubble’s observations, highlighted in yellow in Figure 3.

### 2.2. Image-to-Image Translation

Image-to-image translation [19] is the task of transforming an image from one domain to another, where the goal is to understand the mapping between an input image and an output image. Image-to-image translation methods have shown great success in computer vision tasks, including transferring different styles [20], colorization [21], superresolution [22], visible to infrared translation [23], and many others [24]. There are two types of image-to-image translation methods: *unpaired* [25] (sometimes called unsupervised) and *paired* [26]. Unpaired setups do not require fixed pairs of corresponding images, while paired setups do. In this paper, we also introduce a hybrid method for image-to-image translation, called TURBO [9], which is a generalization of the above-mentioned paired and unpaired setups and provides an information–theoretic interpretation of this method. For the completeness of our study, we also consider newly introduced denoising diffusion probabilistic models (DDPM) as image-to-image translation models [11].

### 2.3. Image-to-Image Translation in Astrophysics

Image-to-image translation has been used in astrophysics for galaxy simulation [3], but these methods have mostly been used for denoising [27] optical and radio astrophysical data [28]. The task of predicting the images of one telescope from another using image-to-image translation remains largely under-researched.

### 2.4. Metrics

The following metrics were used to evaluate the quality of the generated images:

- Mean square error (MSE) between the original and the generated Webb images;
- To address an issue that the MSE is not highly indicative of the perceived similarity of images, we calculate the Structural Similarity Index (SSIM) [12] between the original and generated Webb images;
- Fréchet Inception Distance (FID): proposed in [14]. Instead of a simple pixel-by-pixel comparison of images, FID estimates the mean and standard deviation of one of the deep layers in the pretrained convolutional neural network. It has become one of the most widely used metrics for the image-to-image translation task;
- Peak Signal-to-Noise Ratio (PSNR): This metric evaluates the quality of the generated images by comparing the maximum possible power of a signal (original images) to the power of the same images after distortion (generated images). PSNR is often used as a measure of reconstruction quality in image compression and restoration tasks;
- Learned Perceptual Image Patch Similarity (LPIPS): proposed in [13]. LPIPS measures the perceptual similarity between images by using deep features extracted from a pretrained neural network. It is designed to better reflect human perception of image similarity compared to traditional metrics like MSE or PSNR.

## 3. Proposed Approach

### 3.1. Dataset

We use images from the Hubble and Webb telescopes as the dataset. In particular, we use images of Galaxy Cluster SMACS 0723 [29]. An example of the image is shown in Figure 2. For the Webb, we use post-processed NIRCam images [30], available as RGB images, provided by ESA/NASA/STScI. Webb images are available publicly at [17]. We then select the corresponding Hubble images [18]. Since the Hubble images are smaller than Webb images, we upsampled them using bicubic interpolation for comparison purposes.

### 3.2. Image Registration

**Figure 4:**
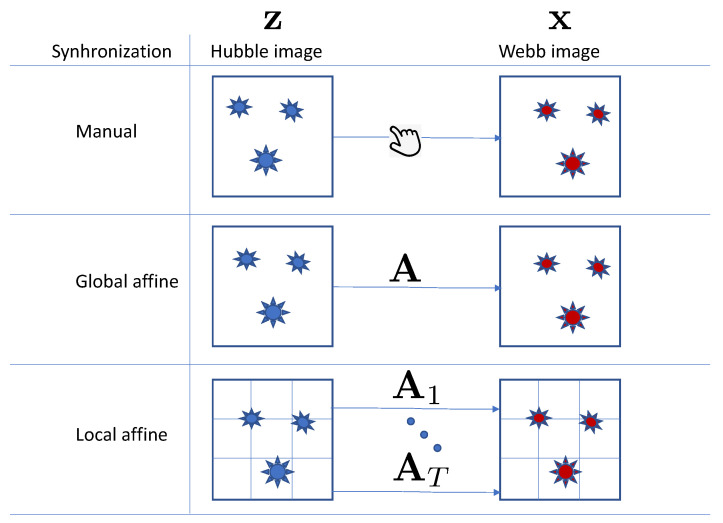
Synchronization setups under investigation in paired image-to-image translation problems: synchronization with respect to celestial coordinates; global synchronization, when images are matched via a global affine transform **A**; and local synchronization, when images are divided into local blocks and matched via a set of local affine transforms $A_{i}$, 1≤i≤T.

Image registration or synchronization is needed to ensure that pixels in different data sources represent the same position in observed space. Even though astronomical data are generally synchronized, there is always room for synchronization improvement, especially at the local level. In this section, we compare three synchronization setups for Hubble to Webb translation: synchronization with respect to celestial coordinates, algorithmic or automated global synchronization, and local synchronization, as schematically shown in Figure 4.

**Synchronization with respect to celestial coordinates**. In this setup, the data are used directly with the provided synchronization with respect to celestial coordinates.

**Global synchronization**. The data are synchronized using SIFT [31] feature descriptors and the RANSAC [32] matching algorithm. The feature descriptors are computed for the entire image from both the Hubble and Webb telescopes.

**Local synchronization**. The data are synchronized using SIFT feature descriptors and the RANSAC matching algorithm, with the feature descriptors being computed from image patches. Specifically, input images from both the Hubble and Webb telescopes are divided into a grid made of nine patches, arranged in a three × three configuration both vertically and horizontally, before the cropping process.

The non-synchronized and synchronized Webb and Hubble images can be viewed in our demo: hubble-to-webb.herokuapp.com (accessed on 8 February 2024).

### 3.3. TURBO

#### 3.3.1. Mathematical Interpretation

**Figure 5:**
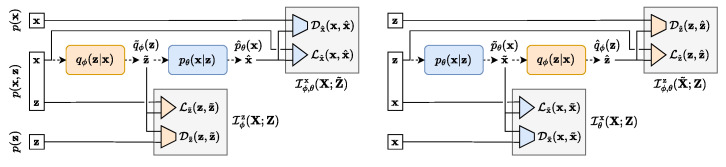
TURBO scheme: direct (**left**) and reverse (**right**) paths.

The TURBO framework [9] is based on an auto-encoder (AE) structure and is represented by an encoder $q_{\phi }\left(z|x\right)$ and a decoder $p_{\theta }\left(x|z\right)$ that are deep networks parametrized by the parameters ϕ and θ, respectively. A block diagram for the TURBO system is shown in Figure 5.

According to the framework we used, given a pair of data samples (Hubble and Webb images) (x,z)∼p(x,z), where z is a Hubble image and x is a Webb image, the system maximizes the mutual information between x and z for both encoder and decoder in *direct* and *reverse* paths.

Two approximations of the joint distribution can be defined as follow:

(1) $$q_{\phi }\left(x,z\right):=q_{\phi }\left(z|x\right)\overset{\begin{array}{c} \mathrm{real}\\ \mathrm{data} \end{array}}{\overset{︷}{p(x)}}=q_{\phi }\left(x|z\right)\overset{\begin{array}{c} \mathrm{synthetic}\\ \mathrm{data} \end{array}}{\overset{︷}{{\tilde{q}}_{\phi }\left(z\right)}},$$

(2) $$p_{\theta }\left(x,z\right):=\underset{\begin{array}{c} \mathrm{known}\\ \mathrm{networks} \end{array}}{\underset{︸}{p_{\theta }\left(x|z\right)}}\;p\left(z\right)=\underset{\begin{array}{c} \mathrm{unknown}\\ \mathrm{networks} \end{array}}{\underset{︸}{p_{\theta }\left(z|x\right)}}\;\;{\tilde{p}}_{\theta }\left(x\right),$$

the marginal distributions are approximated through reparametrizations involving unknown networks. These are represented as ${\tilde{q}}_{\phi }\left(z\right)=\int q_{\phi }\left(x,z\right)dx$ and ${\tilde{p}}_{\theta }\left(x\right)=\int p_{\theta }\left(x,z\right)dz$, relating to the synthetic variables in latent spaces. Furthermore, in our work, we also utilize two approximated marginal distributions for the reconstructed synthetic variables in spaces, denoted as ${\hat{q}}_{\phi }\left(z\right)=\int {\tilde{p}}_{\theta }\left(x\right)q_{\phi }\left(z|x\right)dx$ and ${\hat{p}}_{\theta }\left(x\right)=\int {\tilde{q}}_{\phi }\left(z\right)p_{\theta }\left(x|z\right)dz$.

The variational approximation is considered for the *direct path* of the TURBO system based on the maximization of two bounds on mutual information for the latent space and the reconstruction space:

(3) $$\begin{array}{c} I\left(X;Z\right)=E_{p(x,z)}\left[\log\frac{p(x,z)}{p(x)p(z)}\right]\geq \underset{-L_{\tilde{z}}\left(z,\tilde{z}\right)}{\underset{︸}{E_{p(x,z)}\left[\log q_{\phi }\left(z|x\right)\right]}}-\underset{D_{\tilde{z}}\left(z,\tilde{z}\right)}{\underset{︸}{D_{\mathrm{KL}}\left(p\left(z\right)∥{\tilde{q}}_{\phi }\left(z\right)\right)}}, \end{array}$$

(4) $$\begin{array}{c} I_{\phi }\left(X;\tilde{Z}\right)=E_{q_{\phi }\left(x,z\right)}\left[\log\frac{q_{\phi }\left(x,z\right)}{p\left(x\right){\tilde{q}}_{\phi }\left(z\right)}\right]\geq \underset{-L_{\hat{x}}\left(x,\hat{x}\right)}{\underset{︸}{E_{q_{\phi }\left(x,z\right)}\left[\log p_{\theta }\left(x|z\right)\right]}}-\underset{D_{\hat{x}}\left(x,\hat{x}\right)}{\underset{︸}{D_{\mathrm{KL}}\left(p\left(x\right)∥{\hat{p}}_{\theta }\left(x\right)\right)}}. \end{array}$$

Thus, the network is trained in such a way to maximize a weighted sum of (3) and (4) in order to find the best parameters ϕ and θ of the encoder and the decoder, respectively. This is achieved in the *direct path* by minimising the $L^{\mathrm{direct}}$ loss, representing the left network shown in Figure 5:

(5) $$\begin{array}{c} L^{\mathrm{direct}}\left(\phi ,\theta \right)=L_{\tilde{z}}\left(z,\tilde{z}\right)+D_{\tilde{z}}\left(z,\tilde{z}\right)+\lambda_{D}L_{\hat{x}}\left(x,\hat{x}\right)+\lambda_{D}D_{\hat{x}}\left(x,\hat{x}\right), \end{array}$$

where z is real Hubble image, x is real Webb image, $\tilde{z}$ predicted Hubble image generated by $q_{\phi }\left(z|x\right)$ from real Webb image x, $\hat{x}$ is Webb image reconstructed from generated Hubble image $\tilde{z}$, $L_{\tilde{z}}\left(z,\tilde{z}\right)$ reconstruction loss between real and generated Hubble images, $D_{\tilde{z}}\left(z,\tilde{z}\right)$ discriminator loss for generated Hubble images, $L_{\hat{x}}\left(x,\hat{x}\right)$ present cycle reconstruction loss between real and reconstructed Webb images, $D_{\hat{x}}\left(x,\hat{x}\right)$ is discriminator loss in the reconstructed Webb images, and $\lambda_{D}$ is a parameter controlling the trade-off between the terms in (3) and (4).

The variational approximation for the *reverse path* is:

(6) $$\begin{array}{c} I\left(X;Z\right)=E_{p(x,z)}\left[\log\frac{p(x,z)}{p(x)p(z)}\right]\geq \underset{-L_{\tilde{x}}\left(x,\tilde{x}\right)}{\underset{︸}{E_{p(x,z)}\left[\log p_{\theta }\left(x|z\right)\right]}}-\underset{D_{\tilde{x}}\left(x,\tilde{x}\right)}{\underset{︸}{D_{\mathrm{KL}}\left(p\left(x\right)∥{\tilde{p}}_{\theta }\left(x\right)\right)}}, \end{array}$$

(7) $$\begin{array}{c} I_{\theta }\left(\tilde{X};Z\right)=E_{p_{\theta }\left(x,z\right)}\left[\log\frac{p_{\theta }\left(x,z\right)}{{\tilde{p}}_{\theta }\left(x\right)p\left(z\right)}\right]\geq \underset{-L_{\hat{z}}\left(z,\hat{z}\right)}{\underset{︸}{E_{p_{\theta }\left(x,z\right)}\left[\log q_{\phi }\left(z|x\right)\right]}}-\underset{D_{\hat{z}}\left(z,\hat{z}\right)}{\underset{︸}{D_{\mathrm{KL}}\left(p\left(z\right)∥{\hat{q}}_{\phi }\left(z\right)\right)}}. \end{array}$$

The *reverse path* loss $L^{\mathrm{reverse}}\left(\phi ,\theta \right)$ is represented by the right network shown in Figure 5:

(8) $$\begin{array}{c} L^{\mathrm{reverse}}\left(\phi ,\theta \right)=L_{\tilde{x}}\left(x,\tilde{x}\right)+D_{\tilde{x}}\left(x,\tilde{x}\right)+\lambda_{R}L_{\hat{z}}\left(z,\hat{z}\right)+\lambda_{R}D_{\hat{z}}\left(z,\hat{z}\right), \end{array}$$

where $\tilde{x}$ is a Webb image, generated by $p_{\theta }\left(x|z\right)$ from a real Hubble image z, $\hat{z}$ is a Hubble image reconstructed from generated Webb image $\tilde{x}$, $L_{\tilde{x}}\left(x,\tilde{x}\right)$ is reconstruction loss between the real and generated Webb images, $D_{\tilde{x}}\left(x,\tilde{x}\right)$ is discriminator loss in the generated Webb images, $L_{\hat{z}}\left(z,\hat{z}\right)$ is cycle reconstruction loss between real and reconstructed Hubble images, $D_{\hat{z}}\left(z,\hat{z}\right)$ discriminator loss in the reconstructed Hubble images, and $\lambda_{R}$ is a parameter controlling the trade-off between (6) and (7).

A detailed derivation and analysis of TURBO can be found in [9].

The TURBO method is versatile and adaptable to various setups. It supports a fully paired configuration, utilizing direct and reverse path losses, provided above, which are applicable when data pairs are fully accessible during training. In cases where such pairs are unavailable for training, an unpaired configuration is viable. Additionally, a mixed setup can be employed, combining both paired and unpaired data. This method imposes no constraints on the architecture of the encoder and decoder, offering a broad range of architectural choices.

#### 3.3.2. Paired Setup: Pix2Pix as Particular Case of TURBO

Pix2Pix [7] image-to-image translation method can be viewed as a paired case of TURBO approach, with only reverse path, where $\lambda_{R}=0$ in (9):

(9) $$\begin{array}{c} L^{Pix2Pix}\left(\theta \right)=L_{\tilde{x}}\left(x,\tilde{x}\right)+D_{\tilde{x}}\left(x,\tilde{x}\right) \end{array}.$$

Thus, the direct path is not used as the training of the encoder–decoder pair and Pix2Pix uses uses the deterministic decoder $\tilde{x}=g_{\theta }\left(z\right)$.

#### 3.3.3. Unpaired Setup: CycleGAN as Particular Case of TURBO

The CycleGAN [8] image-to-image translation method can be viewed as a particular case of the TURBO approach, with both a direct and reverse path, with cycle reconstruction losses and discriminator losses for predicted images, with:

(10) $$\begin{array}{c} L^{\mathrm{CycleGAN}\;}\left(\phi ,\theta \right)=D_{\tilde{z}}\left(z,\tilde{z}\right)+\lambda_{D}L_{\hat{x}}\left(x,\hat{x}\right)+\lambda_{T}D_{\tilde{x}}\left(x,\tilde{x}\right)+\lambda_{T}\lambda_{R}L_{\hat{z}}\left(z,\hat{z}\right), \end{array}$$

CycleGAN does not have paired components in the latent space in comparison to TURBO.

### 3.4. Denoising Diffusion Based Image-to-Image Translation

Conditional denoising diffusion probabilistic models [10] for image-to-image translation apply a denoising process that is conditioned on the input image [11]. Image-to-image diffusion models are conditional models of the form $p_{\theta }\left(x|z\right)$, where x is a generated Webb image, and z is a Hubble image, used as a condition. In fact, the DDPM models are derived from the Variational Autoencoder [33] with the decomposition of the latent space of z as a hierarchical Markov model $z_{T}\to z_{T-1}\to \cdots \to z_{0}$ [34].

In practice, the conditional image is concatenated to the input noisy image. During training, detailed in Algorithm 1, we use a simple DDPM training loss (11):

(11) $$\begin{array}{c} L^{DDPM}\left(\theta \right)=E_{t,z,x_{0},\epsilon }\left[{∥\epsilon -\epsilon_{\theta }\left(\sqrt{{\bar{\alpha }}_{t}}x_{0}+\sqrt{1-{\bar{\alpha }}_{t}}\epsilon ,z,t\right)∥}^{2}\right], \end{array}$$

where $x_{0}$ is Webb image, z is the input Hubble image, used in conditioning, ϵ is Gaussian zero mean unit variance noise added at step *t*, $\epsilon_{\theta }$ is conditional DDPM, and ${\bar{\alpha }}_{t}$ is noise scale parameter, added at step *t*.
**Algorithm 1** Training a denoising model $\epsilon_{\theta }$1:Define noise schedule $\beta_{1},\beta_{2},\ldots ,\beta_{T}$2:Compute ${\bar{\alpha }}_{t}$ for t=1 to *T* using ${\bar{\alpha }}_{t}=\prod_{s=1}^{t}\left(1-\beta_{s}\right)$3:**repeat**4:    (x,z)∼p(x,z)5:    ϵ∼N(0,I)6:    t∼1…T7:   Take a gradient descent step on $\nabla_{\theta }{∥\epsilon -\epsilon_{\theta }\left(\sqrt{{\bar{\alpha }}_{t}}x_{0}+\sqrt{1-{\bar{\alpha }}_{t}}\epsilon ,z,t\right)∥}^{2}$8:**until** converged

In the inference phase of the conditional denoising diffusion probabilistic model, detailed in Algorithm 2, the model starts with an initial noisy sample $x_{T}$ from a Gaussian distribution N(0,I); then, the model utilizes a learned denoising function $\epsilon_{\theta }$, which incorporates the conditioning Hubble image x, to iteratively denoise the image at each timestep *t*. The image is updated according to (12):

(12) $$\begin{array}{c} x_{t-1}=\frac{1}{\sqrt{{\bar{\alpha }}_{t}}}\left(x_{t}-\frac{1-{\bar{\alpha }}_{t}}{\sqrt{1-{\bar{\alpha }}_{t}}}\epsilon_{\theta }\left(x_{t},z,t\right)\right)+\sqrt{1-{\bar{\alpha }}_{t}}\epsilon , \end{array}$$

where ϵ is sampled from Gaussian noise. This denoising process is repeated for *T* steps until the final image $x_{0}$ is obtained.
**Algorithm 2** Inference in *T* iterative refinement steps1:$x_{T}∼N\left(0,I\right)$2:**for** t=T,…,1 **do**3:    ϵ∼N(0,I)**if** t>1, **else** ϵ=04:    $x_{t-1}=\frac{1}{\sqrt{{\bar{\alpha }}_{t}}}\left(x_{t}-\frac{1-{\bar{\alpha }}_{t}}{\sqrt{1-{\bar{\alpha }}_{t}}}\epsilon_{\theta }\left(x_{t},z,t\right)\right)+\sqrt{1-{\bar{\alpha }}_{t}}\epsilon$5:**end for**6:**return** $x_{0}$

## 4. Uncertainty Estimation

**Figure 6:**
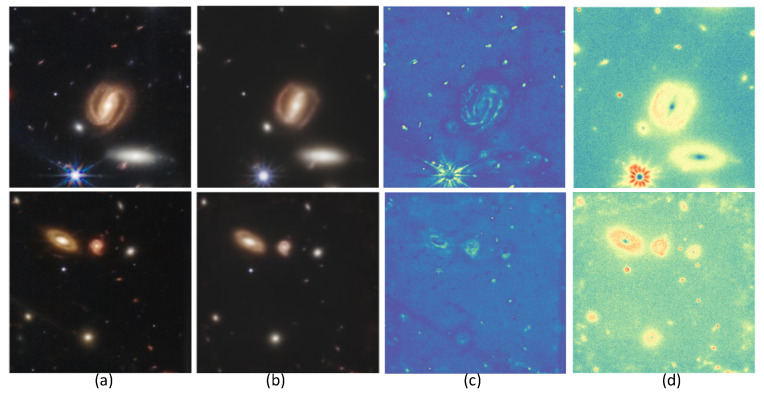
Uncertainty map visualization. (**a**) x target Webb image, (**b**) $\bar{\hat{x}}$ predicted image, averaged from ${\hat{x}}_{i}$, (**c**) true uncertainty, (**d**) estimated uncertainty. The estimated PSUR: 28.99 dB.

In this section, we show how denoising diffusion probabilistic models can be used for the prediction of uncertainty maps. By design, DDPMs are stochastic generators at each sampling step, so it is possible to generate multiple predictions for the same input. The ensemble of predictions allows us to compute the pixel-wise deviation maps that visualize the uncertainty of the predictions. In Figure 6, we display the *true uncertainty map* U, computed as $\sqrt{\frac{\sum_{i=1}^{N}{\left({\hat{x}}_{i}-x\right)}^{2}}{N}}$, where x is the target Webb image, ${\hat{x}}_{i}$ is the *i*-th predicted Webb image, $\bar{\hat{x}}$ is the averaged predicted image estimated from ${\hat{x}}_{i}$, and *N* is the number of generated images. In our experiments, we have used 100 generations to compute the *estimated uncertainty map* $\hat{U}$, computed as $\sqrt{\frac{\sum_{i=1}^{N}{\left({\hat{x}}_{i}-\bar{\hat{x}}\right)}^{2}}{N}}$.

**Figure 7:**
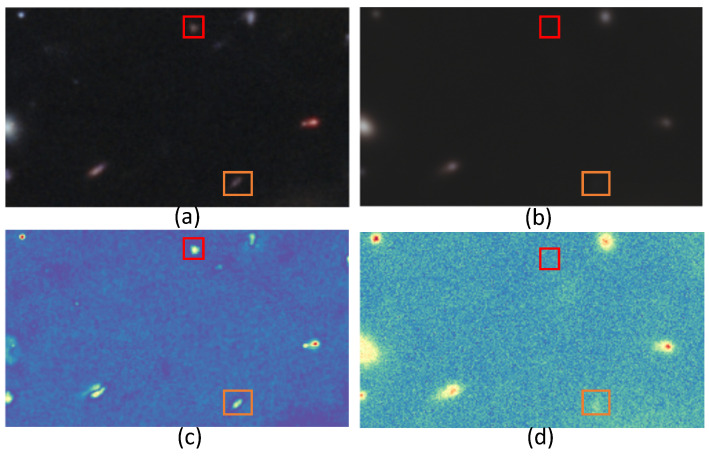
Uncertainty map for point sources: (**a**) target Webb image; (**b**) predicted Webb image; (**c**) true uncertainty; (**d**) estimated uncertainty. The point sources, that were missed, and for which there is no sign in the uncertainty map, are highlighted with a red box. The point sources are missed, but for which there is a sign in the uncertainty map, are highlighted with an orange box. The estimated PSUR: 26.72 dB.

**Figure 8:**
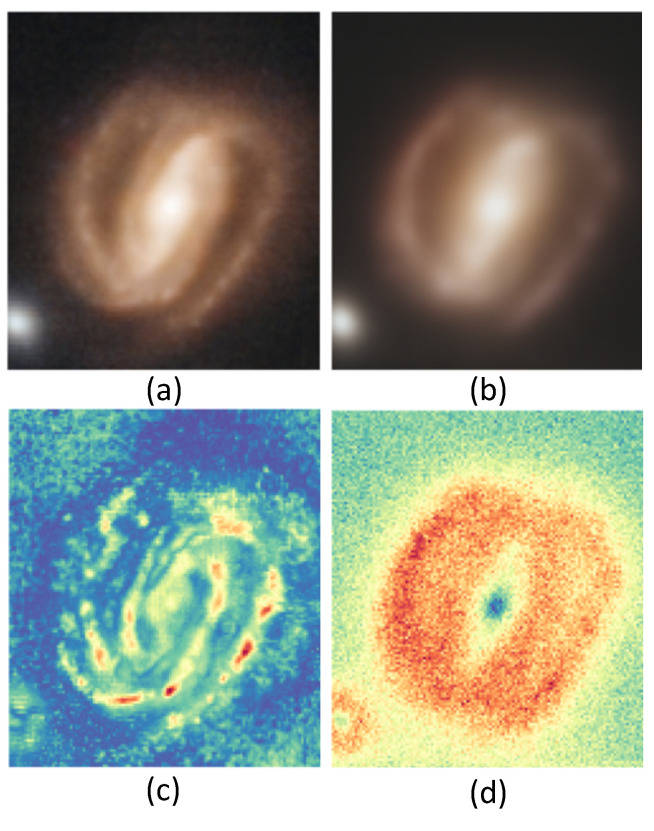
Uncertainty map for the galaxy: (**a**) target Webb image; (**b**) predicted Webb image; (**c**) true uncertainty with respect to the target image; (**d**) estimated uncertainty without the target image that reflects the variability in the generated images. The estimated PSUR: 28.99 dB.

**Figure 9:**
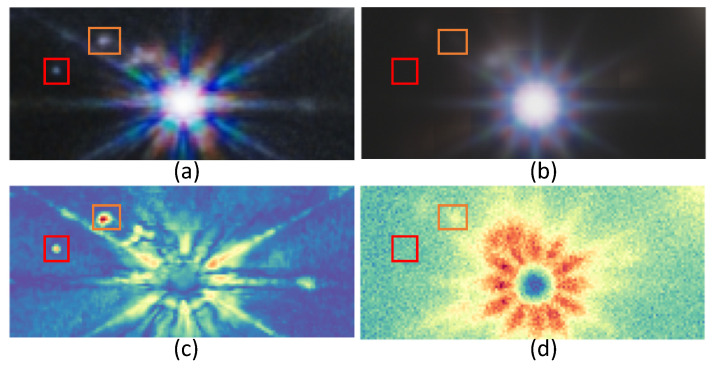
Uncertainty map for the star: (**a**) target Webb image; (**b**) predicted Webb image; (**c**) true uncertainty; (**d**) estimated uncertainty. The point sources, that were missed, and for which there is no sign in the uncertainty map, are highlighted with a red box. The point sources are missed in the predicted Webb, but there is a sign of one in the uncertainty map, which means it was present in some of the predictions. The estimated PSUR: 24.44 dB.

The uncertainty map can be used for analyzing and evaluating the DDPM results by indicating the regions of low and high variability as a measure of uncertainty in each experiment. It is remarkable that this approach is very discriminating for the different types of space objects: point objects (shown in Figure 7, Figure 8 and Figure 9), galaxies (shown in Figure 8), and stars (shown in Figure 9). Furthermore, we have found that the method is able to detect the presence of point source objects in the estimated uncertainty maps, while such objects were not usually directly detectable in the Hubble images or in the predicted Webb images (highlighted with orange boxes in Figure 7 and Figure 9). The point sources that were not present in the Hubble images were not completely predicted in the Webb images when considering these images independently. However, the use of an uncertainty map allowed us to spot their presence in the uncertainty maps, which are highlighted with red boxes in the above-mentioned figures. To further evaluate the performance, we introduce the Peak Signal-to-Uncertainty Ratio (PSUR), computed as $\mathrm{PSUR}=10\cdot \log_{10}\left(\frac{{\mathrm{MAX}}_{x}^{2}}{\mathrm{mean}(\hat{U})}\right)$ dB, where ${\mathrm{MAX}}_{x}$ is the maximum possible pixel value of the image. This metric, analogous to PSNR but using the uncertainty map instead of MSE, offers a measure of how distinguishable the true signal is from the uncertainty inherent in the prediction process. We compute PSUR value for every uncertainty map, shown in Figure 6, Figure 7, Figure 8 and Figure 9.

## 5. Implementation Details

We use PyTorch 1.12 [35] deep learning framework in all our experiments.

**Data.** We use crops from Hubble and Webb images of size 256×256 pixels in each experiment. All of the images used in training and validation are available at github.com/vkinakh/Hubble-meets-Webb, (accessed on 8 February 2024). We apply random horizontal and vertical flipping to each image pair of Hubble–Webb images as augmentation.

**Pix2Pix and CycleGAN.** In the experiments with Pix2Pix and CycleGAN, we use a convolutional architecture consisting of two convolutional layers for downsampling, nine residual blocks, and two transposed convolutional layers for upsampling for both the encoder and decoder. As discriminators, we use PatchGAN [7] with LSGAN loss [36], as provided in the original implementations. During training, we use an Adam [37] optimizer with a learning rate of $2\times 10^{-4}$ and a linear learning rate policy weight decay every 50 steps. Each model is trained for 100 epochs with a batch size of 64. For the experiments, we have used NVIDIA RTX 2080Ti GPU.

**TURBO.** In the experiments with TURBO [9], we use the same convolutional architectures for the encoder and decoder as in the Pix2Pix and CycleGAN experiments. TURBO consists of two convolutional generators: the first, $q_{\phi }\left(x,z\right)$, generates Webb images from Hubble ones, and the second, $p_{\theta }\left(x|z\right)$, generates Hubble images from Webb ones. We use four PatchGAN [7] discriminators: one for generated Webb samples $D_{x\tilde{x}}\left(\tilde{x}\right)$, one for reconstructed Webb samples $D_{x\hat{x}}\left(\hat{x}\right)$, one for generated Hubble images $D_{z\tilde{z}}\left(\tilde{z}\right)$, and one for reconstructed Hubble images $D_{z\hat{z}}\left(\hat{z}\right)$. Alternatively, the TURBO model can only use two discriminators: the first $D_{z}$ for generated and reconstructed Webb images, and the second $D_{x}$ for generated and reconstructed Hubble images. The results using two discriminators are shown in the ablation study in Table 1. As estimation and cycle losses, we use the $\ell_{1}$-metric. We use the LSGAN discriminator loss [36], as in the Pix2Pix and CycleGAN experiments. Similarly, we use the Adam optimizer with a learning rate of $2\times 10^{-4}$ and a linear learning rate policy with decay every 50 steps. The model is trained for 100 epochs with a batch size of 64. For the experiments, we have used NVIDIA RTX 2080Ti GPU.

**DDPM (Palette).** In the experiments, we use a DDPM image-to-image translation model proposed in [11]. We use a UNet [38]-based noise estimator, with self-attention [39]. During training, we use a linear beta schedule with 2000 steps, $10^{-6}$ start, and 0.01 end. During inference, we use a DDPM scheduler with 1000 steps, $10^{-6}$ start, and 0.01 end. The model is trained for 1000 epochs with a batch size of 32. For the experiments, we have used NVIDIA A100 GPU.

During inference, since our images exceed 256×256 pixels, we employ a method known as *stride prediction* to predict patches of size 256×256 using a selected stride value. This method works systematically across the image: starting from the top-left corner at position (0,0), we predict the first patch, then move horizontally by stride *s* to predict the next, proceeding row by row until the entire image is covered. If the bottom or right edge is reached, the next row begins just below the starting point or back at the left edge, respectively. After predicting all patches, we save the images and track the prediction count for each pixel. The final pixel value is determined by averaging across all predictions for that pixel, ensuring a seamless image reconstruction.

## 6. Results

In this section, we report image-to-image translation results for the prediction of Webb telescope images based on Hubble telescope images. In Table 2, we report results for four setups: (a) unpaired setup; (b) paired setup with the synchronization with respect to celestial coordinates, where images were synchronized by hand; (c) paired setup with global synchronization, where the full image was synchronized using a single affine transform; and (d) paired setup with local synchronization, where the images were split into multiple patches and then each of the Hubble and Webb patches were synchronized individually. For each setup, we have defined a training set that covers approximately 80% of the input image of the galaxy clusters SMACS 0723, and the rest is used as a validation set for results. We make sure that the training and validation set cover different parts of the sky and never overlap even for a single pixel. When generating images for evaluation, since the validation images are larger than 256×256, we have used the stride prediction described above with a stride of four. It is shown in Table 2 that the synchronization of the data is very important, as all of the considered models perform best when the data are locally synchronized. This fact was not well addressed in previous studies, to the best of our knowledge. Also, we show that the DDPM-based image-to-image translation model outperforms the CycleGAN, Pix2Pix, and TURBO models in terms of MSE, SSIM, PSNR, FID and LPIPS metrics. The only downside of the DDPM model is its inference time, which is 1000 times longer than the inference time of Pix2Pix, CycleGAN and TURBO. This might be a serious limitation in practice, considering the size and number of astronomical images.

In Table 3, we compare parameter counts and inference times for a 256 × 256 image from the models considered in the study. The DDPM model is particularly noteworthy for its extensive parameter count, with both trainable and inference parameters reaching 62.641 Mio. It also necessitates 1000 generation steps, contributing to a longer inference time of approximately 42.77 s. Conversely, Pix2Pix, CycleGAN, and Turbo demonstrate a more streamlined parameter structure. These models employ generators with a uniform parameter count of 11.378 Mio and discriminators with 2.765 Mio parameters. Pix2Pix operates with one generator and one discriminator, CycleGAN with two of each, and Turbo with two generators and four discriminators. Despite the architectural differences, these models maintain compact trainable parameters, ranging from 14.143 Mio to 33.816 Mio, and achieve notably swift inference times, clocked at around 0.07 s. The inference time is averaged over 100 generations for each model on a single RTX 2080 Ti GPU with a batch size of one.

In Table 1, we perform ablation studies on the paired TURBO and Pix2Pix image-to-image translation models. We compare these models trained under various conditions: (a) with the $L_{1}$ loss, which is the mean absolute error, (b) with the $L_{2}$ loss, which is the mean squared error, (c) with both $L_{1}$ and $L_{2}$ losses, (d) with $L_{1}$ loss and the Learned Perceptual Image Patch Similarity (LPIPS) loss using a VGG encoder [13]. We also explore Pix2Pix configurations, such as Pix2Pix with $L_{1}$ loss plus a discriminator, Pix2Pix combined with LPIPS loss and a VGG encoder, along with variations of the TURBO model: TURBO with LPIPS loss, TURBO operating only in reverse pass, and TURBO using the same discriminator for both generated and reconstructed images. Models are trained and evaluated on data synchronized locally. As Table 1 indicates, Pix2Pix models and those without a discriminator perform better on paired metrics (MSE, PSNR, SSIM), whereas TURBO-based methods excel in image quality metrics (LPIPS, FID). Notably, the DDPM-based image-to-image translation method outperforms other methods discussed in the ablation study.

## 7. Conclusions

In this paper, we have proposed the use of image-to-image translation approaches for sensor-to-sensor translation in astrophysics for the task of predicting Webb images from Hubble. The novel TURBO framework serves as a versatile tool that outperforms existing GAN-based image-to-image translation methods, offering better quality in generated Webb telescope imagery and information-theoretic explainability. Furthermore, the application of DDPM for uncertainty estimation introduces a probabilistic dimension to image translation, providing a robust measure of reliability previously unexplored in this context. We show the importance of synchronization in paired image-to-image translation approaches.

This research not only paves the way for improved astronomical observations by leveraging advanced computational techniques but also advocates for the application of these methods in other domains where image translation and uncertainty estimation are crucial. As we continue to venture into the cosmos, the methodologies refined here will undoubtedly become instrumental in interpreting and maximizing the utility of the data we collect from advanced telescopes.

## 8. Future Work

Out future research will include an approach to refine and enhance the methodologies discussed in this paper. A particular focus will be directed towards improving the TURBO model, which, while being computationally efficient, currently lags behind DDPM in terms of performance. TURBO model improvement will be mostly focused on architectural improvements of generators. In parallel, we plan to undertake a thorough investigation into the resilience of our applied methods against various data preprocessing techniques, including different forms of interpolation. This study aims to ensure the robustness and adaptability of our models across a spectrum of data manipulation scenarios. Moreover, the exploration of existing sampling techniques within DDPMs will be pursued with the goal of expediting inference times. This focus is expected to significantly improve the models’ efficiency, rendering them more suitable for real-time applications.

The current research specifically focuses on the analysis of RGB pseudocolor images. A significant portion of our future work will be dedicated to the meticulous training and evaluation of the proposed models on raw astrophysical data. This will involve the integration of specialized astrophysical metrics designed to align with the unique properties of such data, thereby assuring that our models are not only statistically sound but also truly resonate with the practical demands and intricacies of astrophysical research. We aspire to bridge the gap between theoretical robustness and real-world applicability, setting the stage for transformative developments in the field of image-to-image translation in astrophysical data analysis. 

## Figures and Tables

**Table 1:** Ablation studies on paired models Pix2Pix and TURBO on locally synchronized data. All results are obtained on Galaxy Cluster SMACS 0723. The label “TURBO same *D*” corresponds to an approach, when the same discriminator is used for generated and reconstructed Webb and Hubble images. The label “LPIPS” denotes adding perceptual similarity loss.

Method	MSE ↓	SSIM ↑	PSNR ↑	LPIPS ↓	FID↓
$L_{1}$	0.002	0.93	26.94	0.47	83.32
$L_{2}$	0.002	0.93	26.98	0.47	76.03
$L_{1}+L_{2}$	0.002	0.93	26.93	0.47	82.71
$L_{1}+$ LPIPS	0.002	0.93	26.68	0.44	72.84
Pix2Pix	0.002	0.93	26.78	0.44	54.58
Pix2Pix + LPIPS	0.003	0.93	27.02	0.44	58.86
TURBO	0.003	0.92	25.88	0.41	43.36
TURBO + LPIPS	0.003	0.92	25.91	0.39	50.83
$L^{\mathrm{reverse}}$	0.002	0.93	26.15	0.45	70.51
$L^{\mathrm{reverse}}$ + LPIPS	0.002	0.93	26.13	0.46	67.52
TURBO same *D*	0.002	0.92	26.04	0.4	55.29
TURBO same *D* + LPIPS	0.002	0.92	26.13	0.39	55.88

**Table 2:** Hubble-to-Webb results. All results are obtained on a validation set of Galaxy Cluster SMACS 0723.

Method	MSE ↓	SSIM ↑	PSNR ↑	LPIPS ↓	FID ↓
**unpaired**					
CycleGAN	0.010	0.83	20.11	0.48	128.12
**paired: synchronization with respect to celestial coordinates**					
Pix2Pix	0.007	0.87	21.37	0.5	102.61
TURBO	0.008	0.85	20.87	0.49	98.41
DDPM (Palette)	0.003	0.88	25.36	0.43	51.2
**paired: global synchronization**					
Pix2Pix	0.003	0.92	25.85	0.46	55.69
TURBO	0.003	0.91	25.08	0.45	48.57
DDPM (Palette)	0.002	0.94	28.12	0.45	43.97
**paired: local synchronization**					
Pix2Pix	0.002	0.93	26.78	0.44	54.58
TURBO	0.003	0.92	25.88	**0.41**	43.36
**DDPM** **(Palette)**	**0.001**	**0.95**	**29.12**	0.44	**30.08**

**Table 3:** Analysis of parameter complexity and inference time in image-to-image translation models.

Model	Trainable Params	Inference Paras	Inference Time
DDPM (1000 steps)	62.641 Mio	62.641 Mio	42.77 ± 0.18 s
Pix2Pix	14.143 Mio	11.378 Mio	0.07 ± 0.004 s
CycleGAN	28.286 Mio	11.378 Mio	0.07 ± 0.004 s
TURBO	33.816 Mio	11.378 Mio	0.07 ± 0.004 s

## Data Availability

The code and data used in the study can be accessed at public repository: github.com/vkinakh/Hubble-meets-Webb, (accessed on 8 February 2024). The experimental results can be accessed at hubble-to-webb.herokuapp.com, (accessed on 8 February 2024).

## Abbreviations

The following abbreviations are used in this manuscript:

GAN	Generative adversarial network
DDPM	Denoising diffusion probabilistic model
MSE	Mean squared error
PSNR	Peak signal to noise ratio
LPIPS	Learned perceptual image patch similarity
FID	Fréchet inception distance
RGB	Red, green, blue
AE	Auto-encoder
SSIM	Structural similarity index measure
TURBO	Two-way Uni-directional Representations by Bounded Optimisation
HST	Hubble Space Telescope
JWST	James Webb Space Telescope
LSGAN	Least Squares Generative Adversarial Network
SIFT	Scale-Invariant Feature Transform
RANSAC	Random Sample Consensus
ESA	European Space Agency
NASA	National Aeronautics and Space Administration
STScI	Space Telescope Science Institute
